# Optimization of DNA Recovery and Amplification from Non-Carbonized Archaeobotanical Remains

**DOI:** 10.1371/journal.pone.0086827

**Published:** 2014-01-27

**Authors:** Nathan Wales, Kenneth Andersen, Enrico Cappellini, María C. Ávila-Arcos, M. Thomas P. Gilbert

**Affiliations:** 1 Department of Anthropology, University of Connecticut, Storrs, Connecticut, United States of America; 2 Centre for GeoGenetics, University of Copenhagen, Copenhagen, Denmark; 3 Department of Environment and Agriculture, Curtin University, Perth, Western Australia, Australia; Institut de Biologia Evolutiva - Universitat Pompeu Fabra, Spain

## Abstract

Ancient DNA (aDNA) recovered from archaeobotanical remains can provide key insights into many prominent archaeological research questions, including processes of domestication, past subsistence strategies, and human interactions with the environment. However, it is often difficult to isolate aDNA from ancient plant materials, and furthermore, such DNA extracts frequently contain inhibitory substances that preclude successful PCR amplification. In the age of high-throughput sequencing, this problem is even more significant because each additional endogenous aDNA molecule improves analytical resolution. Therefore, in this paper, we compare a variety of DNA extraction techniques on primarily desiccated archaeobotanical remains and identify which method consistently yields the greatest amount of purified DNA. In addition, we test five DNA polymerases to determine how well they replicate DNA extracted from non-charred ancient plant remains. Based upon the criteria of resistance to enzymatic inhibition, behavior in quantitative real-time PCR, replication fidelity, and compatibility with aDNA damage, we conclude these polymerases have nuanced properties, requiring researchers to make educated decisions as to which one to use for a given task. The experimental findings should prove useful to the aDNA and archaeological communities by guiding future research methodologies and ensuring precious archaeobotanical remains are studied in optimal ways, and may thereby yield important new perspectives on the interactions between humans and past plant communities.

## Introduction

Ancient DNA (aDNA) studies have become an integral part of Quaternary research, providing invaluable anthropological and biological insights, on issues as diverse as human evolution [1], modern human migrations [2]–[4], plant and animal domestication [5], [6], and paleoecology [7]. Research on plant aDNA from archaeological contexts is of particular interest because archaeobotanical remains can provide important data on subsistence patterns, human behavioral variability, domestication, and broader environmental issues [8]–[10]. Despite this rich potential, relatively few researchers have studied aDNA from plant materials [9], [11]; the scarcity of this line of research can be partially attributed to the many methodological challenges posed by ancient plant materials.

In addition to the issues of contamination and biomolecular degradation faced by all aDNA research [12], ancient plant materials frequently contain compounds that impede DNA extraction and enzymatic reactions, including the polymerase chain reaction (PCR). In modern plant materials, polysaccharides and polyphenols, such as tannins, pose significant problems for the extraction of nucleic acids [13]; these compounds may still thwart geneticists millennia after the death of a plant. In addition, archaeological plant materials are often rich in humic acids, some of which originate from associated sediments. These darkly-pigmented compounds are often inadvertently extracted together with DNA and inhibit many DNA polymerases which are required for genetic analyses [14]. Even when DNA eluates are visually transparent, inhibitors may still be present, leading to PCR failures.

In their systematic review of aDNA techniques, Rohland and Hofreiter [15] explore numerous protocols, the use of different binding salts, incubation modifications, PCR additives, and DNA polymerases. The results of the study have been influential in the aDNA community and have been adopted by a number of researchers, including for the prominent Neanderthal genome project [1]. Nevertheless, Rohland and Hofreiter’s [15] investigation focused only upon aDNA from bones, and therefore the findings may not be applicable to other aDNA source materials, including ancient plant remains. In this article, we expand upon Rohland and Hofreiter’s [15] work by examining the effectiveness of various extraction techniques on non-charred archaeobotanical remains and the relative capabilities of different polymerases to amplify aDNA. Given the growing importance of high-throughput sequencing (HTS) technologies in plant aDNA research [9], issues and goals related to HTS are given special attention.

### Goals for aDNA Extractions

The fundamental aim of DNA extractions of archaeobotanical remains is to isolate as much endogenous DNA from a sample as possible. Ancient samples characteristically have few copies of endogenous DNA, and these molecules are usually fragmented into segments less than a few hundred base pairs (bp) in length [16]. Optimizing aDNA recovery has become even more important in the era of HTS [17]. For conventional PCR-based studies, it is only necessary for the locus of interest to be amplified, and amplification can theoretically initiate from a single template molecule. HTS, on the other hand, require a much larger “library” of DNA molecules (that is, DNA molecules from a sample with special nucleotide adapters attached to each end). HTS platforms require libraries to be amplified to a specified starting concentration, and if DNA extract concentrations are low, more amplification cycles are required, leading to PCR drift and clonality [18], [19].

While it is important to extract as much DNA from an ancient sample as possible, the DNA must also be relatively pure: clear of other cellular components like proteins and lipids that might otherwise hinder downstream analyses. For archaeobotanical remains in particular, it is vital to remove substances which impair enzymatic reactions, including humic acids and polyphenols.

### Goals for Polymerases in aDNA Amplification

Ideally all traces of inhibitory substances would be removed in the course of DNA extraction; however, in some instances these substances remain, often leaving DNA eluates pigmented [20]. Such recalcitrant samples presumably contain humic acids and DNA strands of the same molecular weight, and these molecules consequently coprecipitate in purifications due to their shared anionic properties [21]. Repeated purifications using silica and other methods have been investigated [22]–[24], but since every additional purification step can reduce DNA yield, and because PCR inhibitors may not manifest themselves as obvious pigmentation, it is advantageous to use polymerases that tolerate residual inhibitors.

Real-time quantitative PCR (qPCR) experiments have been designed to study DNA from archaeobotanical remains [25], but there has been little research into the compatibility of different polymerases and PCR additives in qPCR. Exploratory experimentation (N. Wales, unpublished data) suggested that some polymerases do not exhibit normal amplification curves when samples are pigmented or when certain PCR additives are included in the reaction. As departures from ideal amplification curves may lead to inaccurate DNA quantification, it is important to know which polymerases yield consistent qPCR results under a broad range of conditions.

The fidelity of polymerases is an important concern, especially when aDNA libraries are amplified for HTS. Ancient samples frequently yield low levels of coverage for all loci, making it challenging to identify which genetic motif is real and which is the result of polymerase copy errors.

The degraded and damaged nature of aDNA has a profound effect on the performance of polymerases. In particular, research has identified cytosine deamination, a hydrolysis reaction in which cytosine is converted to uracil, as the main source of the problem [26]–[28]. The presence of uracil in aDNA molecules has adverse effects in PCR because DNA polymerases cannot add the appropriate complementary nucleotide to the opposite DNA strand. Instead, polymerases either 1) stop replicating the DNA molecule, or 2) insert adenine which is complementary to uracil in RNA. The latter scenario leads to an apparent C-to-T transition in the template molecule [28], [29]. Depending on the research goals, either of the available options may be preferable. For example, if a polymerase does not copy damaged DNA molecules, bioinformatic analyses are simplified as it can be assumed that damage is not a factor in generating sequence variation. On the other hand, if nearly all molecules are damaged, the polymerase may fail to amplify anything, thus providing no data at all. Additionally, by using a polymerase which pairs uracil with adenine, one may argue for the authenticity of aDNA based upon damage patterns [30], [31]. It is therefore important to be fully aware of how a given polymerase handles damage.

## Materials and Methods

The authors thank the following researchers for permission for destructive analysis of archaeobotanical remains: Boris Gasparyan, Institute of Archaeology and Ethnology, National Academy of Sciences, Yerevan, Armenia; Giovanna Bosi and Anna Maria Mercuri, Museo Di Paleobiologia e dell’Orto Botanico, Università di Modena e Reggio Emilia, Modena, Italy; Girolamo Fiorentino, Dipartimento di Beni Culturali, University of Salento, Lecce, Italy; Mike Jacobs, Arizona State Museum; and José Luis Punzo-Díaz, Instituto Nacional de Antropología e Historia, Centro INAH Michoacán, Mexico.

### Comparison of Extraction Methods

All extractions and PCR setups were performed in a dedicated clean laboratory at the University of Copenhagen, which conforms to the highest standards for the field [32]. Methodological experiments on plant aDNA are fundamentally complicated by limited numbers of suitable specimens and potentially variable DNA preservation among samples, however extractions were designed to minimize variability within a collection of samples. Over three rounds of experiments, sets of archaeobotanical remains were extracted using three to five different methods, and tested for DNA yield and purity. We refer to the methods according to the leading author of the first publication to describe the technique or the commercial name, as listed in Table 1. Appendix S1 provides detailed protocols for all methods, including any modifications from the authors’ or manufacturers’ specifications.

**Table 1:** Extraction techniques compared in this study.

Experiment phase	Name	Method synopsis and relevant information	Reference
Phase 1	Epicentre	QuickExtract Plant DNA Extraction Solution. Designed to extract DNA frommodern plant remains in 10 minutes.	Epicentre, Madison, WI
	Finnzymes	Phire Plant Direct PCR kit. Sample incubated for 3 minutes in buffer andimmediately amplified.	Thermo Fisher Scientific, Waltham, MA
	Gilbert	Digestion in SDS, DTT, and Proteinase K, followed by phenol and chloroform extraction. Previously used to extract DNA from ancient grapes [58].	Gilbert *et al.* [37]
	Japelaghi	Digestion in PVP, CTAB, and 2-mercaptoethanol followed by chloroform-isoamylalcohol extraction. Method designed for modern plant remains rich intannins.	Japelaghi *et al.* [13]
	MO BIO	PowerLyzer PowerSoil DNA Isolation kit. Used to recover aDNA from humic-richsoils [43], [59].	MO BIO Laboratories, Carlsbad, CA
Phase 2^1^	Gilbert	See phase 1.	See phase 1.
	Palmer	Digestion in CTAB, followed by chloroform-isoamyl alcohol extraction, andpurification in Qiagen MinElute column.	Modified from Palmer *et al.* [39]
	Rohland	Digestion in SDS, DTT, and Proteinase K, followed by DNA binding to silicapellet. Silica extraction previously found to be optimal for extracting aDNAfrom bones.	Modified from Rohland and Hofreiter [15]
Phase 3	Andersen	Digestion in 2-mercaptoethanol, DTT, and Proteinase K, followed by MOBIOinhibitor removal, phenol and chloroform extraction, and Millipore filterpurification. Designed to recover aDNA from sediment.	Experimental method developed by Kenneth Andersen
	Gilbert	See phase 1.	See phase 1.
	Palmer	See phase 2, but with purification in Millipore filter and Qiagen DNeasy silicacolumn. Exact method used to recover aDNA from ancient barleyremains [39].	Palmer *et al.* [39]

1 Extraction methods in phase 2 were conducted in three ways: according to the specified directions, with MO BIO C2 and C3 solutions added before extraction, and with MO BIO C2 and C3 solutions used after extraction. See the text and Appendix S1 for further details.

Archaeobotanical remains from a variety of contexts were extracted, listed in Table 2. When deemed sufficiently intact, seeds were cleaned in 0.5% bleach (NaClO) and rinsed in molecular grade water before being extracted; seeds with small cracks or other imperfections, indicated in Table 2, were instead wiped with a towel. The cleaning of other types of archaeobotanical remains, such as maize cobs and grape branches, was conducted by removing exterior surfaces with sterile tools. Most archaeobotanical remains were desiccated, although one set was waterlogged. No charred archaeobotanical remains were tested in these experiments because burned remains often contain little or no endogenous DNA that can be amplified by PCR [33]–[35]. This is an important consideration because macrobotanical remains are most frequently preserved at archaeological sites through charring or carbonization [36]. Desiccation and waterlogging are comparatively less common processes by which plant remains become preserved; nonetheless, desiccated and waterlogged macrobotanicals have been recovered from archaeological sites around the world and are much more likely to contain endogenous aDNA since they have not been exposed to high temperatures. Thus, these experiments are most pertinent to non-charred remains, although some findings may prove applicable to charred remains in subsequent analyses.

**Table 2:** Archaeobotanical remains analyzed.

Sample information			Archaeological context				Extraction phase			PCR tests
Name	Species	Tissue^1^	Site	Geographic location	Repository^2^	Provenience and age	1	2	3	
ARE-A	*Vitis vinifera*	Pips	Areni-1	Areni, Armenia	IAE	Trench 1, square P30/31, locality 2,spit 6. Medieval context.	X			
ARG	*Vitis vinifera*	Pips	Fossato	Argenta (FE), Italy	UMeRE	SU^3^ 2.2. 1275–1325 A.D.	X			
CPR-A^4^	*Vitis vinifera*	Pips	Corso Porto Reno –Via Vaspergolo	Ferrara, Italy	UMeRE	SU 1703. Medieval context.	X			
LUG	*Vitis vinifera*	Pips	Piazza Baracca	Lugo (RA), Italy	UMeRE	SU 144. 15^th^–16^th^ c. A.D.	X			Polymerase fidelity
PAR-A	*Vitis vinifera*	Pips	Piazza Municipale	Parma, Italy	UMeRE	SU 320. 4^th^–2^nd^ c. B.C.	X			
SAM^4^	*Vitis vinifera*	Pips	Pozzo 1 Domagnano	San Marino, Republic of San Marino	UMeRE	SU 565. Late Roman–Gothiccontext.	X			
VAD-A	*Vitis vinifera*	Pips	Vasca Ducale PiazzaMunicipale	Ferrara, Italy	UMeRE	SU 1050. 2^nd^ half 15^th^ c. A.D.	X			Polymerase fidelity
CAS	*Cornus mas*	Seeds	Cassa di Risparmio	Modena, Italy	UMeRE	SU 31. Roman context.		X		
SAF	*Vitis vinifera*	Pips	Piazzale SanFrancesco	Modena, Italy	UMeRE	SU 16. 10^th^–11^th^ c. A.D.		X		
ARE-B^5^	*Gossypium* sp.	Seeds	Areni-1	Areni, Armenia	IAE	Trench 1, square K35, spit 4.Medieval context.		X	X	Inhibition
VAD-B	*Olea europaea*	Pits	Vasca Ducale PiazzaMunicipale	Ferrara, Italy	UMeRE	SU 1050. 2^nd^ half 15^th^ c. A.D.		X	X	Polymerase fidelity
ARE-C^5^	*Vitis vinifera*	Branch	Areni-1	Areni, Armenia	IAE	Trench 1, square N16, locality 29.Medieval context.			X	
CDM^5^	*Zea mays*	Cob	Cueva del Maguey 1	Pueblo Nuevo, Durango, Mexico	INAH	Specimen ID: 10189. 1410±25 ^14^CYBP			X	
CPR-B	*Cornus mas*	Seeds	Corso Porta Remo-ViaVespergolo	Ferrara, Italy	UMeRE	SU 2597. Medieval context.			X	
PAR-B	*Vitis vinifera*	Pips	Piazza Municipale	Parma, Italy	UMeRE	SU 165. Medieval context.			X	
SUP^4^	*Vitis vinifera*	Waterlogged pips	Loc. Scorpo	Supersano (LE), Italy	US	Excavated from well. 7^th^–8^th^ c. A.D.			X	
THR	*Zea mays*	Kernels	Turkey House Ruin	Navajo County, Arizona	ASM	Specimen ID: 935. 723±23^14^CYBP			X	
SPC	*Vitis vinifera*	Pips	Via San Pietro	Modena, Italy	UMeRE	Excavated from compostingfeature. Medieval.				Inhibition

1 Tissues are desiccated except where noted.

2 Samples provided by archaeologists and curators, as listed in acknowledgements. ASM: Arizona State Museum; IAE: Institute of Archaeology and Ethnology, National Academy of Sciences, Yerevan, Armenia; INAH: Instituto Nacional de Antropología e Historia, Centro INAH Michoacán, Mexico; UMeRE: Università di Modena e Reggio Emilia, Modena, Italy; US: University of Salento, Lecce, Italy.

3 SU: Stratigraphic unit.

4 Seeds cleaned by wiping with a dry paper towel. All other seeds cleaned by washing in 0.5% bleach.

5 Samples cleaned by removing exterior (seed coat or bark) with sterile scalpel.

In extraction phase 1, seven sets of *Vitis vinifera* pips were extracted. Grapes were tested because they contain a number of PCR inhibitors and provide a challenge even for genetic studies of modern material [13]. In extraction phase 1, a single seed was extracted with a given method. Recognizing that DNA within samples may be differentially preserved, phases 2 and 3 were conducted on a homogenized collection of seeds from a given context, thereby standardizing the amount of aDNA, contaminant DNA, and inhibitory substances. In addition, a wider range of species and contexts were tested in later extraction phases: four sets of archaeobotanical remains were tested in phase 2 and eight sets in phase 3.

In phase 1, we compared five extraction techniques which have been designed for either ancient materials or modern plant remains. Samples were tested in duplicate for the Gilbert *et al.* [37], Japelaghi *et al.* [13], and MO BIO methods; however, due to a limited number of seeds from identical contexts, it was not possible to perform duplicate extractions for the Epicentre and Finnzymes techniques. Extraction methods were compared on the basis of three criteria: DNA concentration measured on a Qubit 1.0 Fluorometer (Invitrogen, Carlsbad, CA), sample purity measured on a NanoDrop 1000 spectrophotomer (Thermo Scientific, Waltham, MA), and amplification success for the ribulose-bisphosphate carboxylase (*rbcL*) gene, a universal plant marker [38]. PCR conditions for the *rbcL* locus are listed in Appendix S1.

The most promising method was advanced to phase 2, where it was compared with Palmer *et al.*’s [39] extraction method (with minor modifications as listed in Table 1) for ancient plants and a silica pellet extraction, the top performing technique in Rohland and Hofreiter’s [15] study on isolating aDNA from bones. In addition to conducting the extractions according to the specified directions, the methods were modified with the addition of MO BIO ‘C2’ and ‘C3’ solutions (MO BIO Laboratories, Carlsbad, CA), reagents designed to precipitate humic acids and increase sample purity. This modification was conducted either after overnight incubation in digestion buffer or directly after DNA extraction. The same criteria were used to compare the methods as in phase 1, with the addition of sequencing *rbcL* products to determine if endogenous DNA was recovered.

For phase 3, the two top performing methods were further compared, along with an experimental technique developed by one of the authors of this paper (KA). This method, referred to as the Andersen method, is part of an ongoing project to extract aDNA from sediments, and therefore may not be fully optimized. Nonetheless, preliminary findings suggest the Andersen method readily handles humic-rich sediments, and it was hypothesized the technique may also effectively isolate DNA from archaeobotanical remains. In addition to the above previously used testing criteria, the three methods were compared using a qPCR assay for the *rbcL* generic marker to more precisely determine the amount of plant DNA recovered (for details, see “qPCR assay for quantifying DNA in extraction phase 3″ in Appendix S1). This approach was deemed necessary because pigmentation in some extracts could lead to erroneous DNA concentration readings in the Qubit Fluorometer.

### Comparison of DNA Polymerases

Enzymatic inhibition of five polymerases was tested by amplifying exogenous tiger (*Panthera tigris*) DNA “spiked” into pigmented plant eluates. As indicated in Table 2, heavily pigmented DNA extracts from two ancient plant samples were used as inhibiting substances: medieval cotton (*Gossypium* sp.) seeds from the Areni-1 site in Areni, Armenia, and medieval grape (*Vitis vinifera*) pips from the Via San Pietro site in Modena, Italy. Varying amounts of inhibiting solutions were added to PCR reactions, with pigmented extracts representing up to 40% of the reaction volume. Polymerases were selected based upon either their ubiquity in aDNA research, advertised fidelity, or purported ability to overcome inhibition, as summarized in Table 3. PCR details for each polymerase are located in Table S1. As bovine serum albumin (BSA) has been shown to prevent inhibition and increase the likelihood of amplification success in ancient samples [15], [40], reactions were conducted with and without 0.8 mg/mL BSA additive.

**Table 3:** Polymerases tested.

Polymerase	Vendor	Notable features
AmpliTaq Gold	Applied Biosystems, Foster City, CA	Commonly used in aDNA research
Omni Klentaq	DNA Polymerase Technology, St. Louis, MO	Engineered to overcome multiple sources of inhibition, including blood and soil
PfuTurbo C_x_ Hotstart	Agilent Technologies, La Jolla, CA	Purportedly reads through uracil while maintaining high fidelity
Phire Hot Start II	Finnzymes (Thermo Fisher Scientific), Waltham, MA	Designed to overcome inhibition and features rapid processivity
Phusion Hot Start	Finnzymes (Thermo Fisher Scientific), Waltham, MA	Engineered for high fidelity and rapid processivity

AmpliTaq Gold, Omni Klentaq, and PfuTurbo C_x_ Hotstart were further tested for potential use in qPCR assays by amplifying spiked DNA in varying concentrations of inhibitors. Experimentation suggested that BSA occasionally interfered with the detection of fluorescence with AmpliTaq Gold; therefore, for each polymerase, reactions were conducted with and without 0.8 mg/mL BSA. The effects of BSA and inhibition were observed through changes in cycle threshold (C_t_) and amplification curves. Experiment conditions are listed in Appendix S1 in the section “qPCR inhibition testing.”

Polymerase fidelity and compatibility with aDNA damage were investigated through “deep sequencing” of an endogenous DNA marker from ancient plant samples. This approach is commonly used to characterize biodiversity in environmental samples [41], including ancient ones [42]. In such studies, a universal genetic marker for a group of organisms, such as plants or animals, is amplified and sequenced on a HTS platform to identify all species present in the sample and their relative proportions [43]. Here, the aim of deep sequencing is to test thousands of copies of the plant *rbcL* marker amplified from a sample to infer how often polymerases make errors. PCR products from three ancient plant samples, listed in Table 2, were sequenced on a Roche/454 Genome Sequencer FLX platform (for further information, see “Deep sequencing of *rbcL* products” in Appendix S1). Reads were aligned to the expected sequence in Geneious Pro 5.5.7 [44] and nucleotide misincorporations, insertions, and deletions were analyzed.

## Results

### Extraction Comparisons

#### Phase 1

The five extraction methods yielded highly variable DNA concentrations, amplification success rates, and purity levels. The Epicentre and Finnzymes extraction methods frequently yielded DNA eluates that were darkly pigmented. This is significant because DNA concentrations, as measured on the fluorometer, could produce anomalous readings if the pigmented eluates prevent accurate assessment of DNA-binding dyes. Therefore, the primary indication of success was taken to be the rate of successful amplification of genetic plant markers. Based on this criterion, the Gilbert method was the top performer, with successful amplification of the *rbcL* marker in 10 out of 14 specimens, as listed in Table 4. Japelaghi’s method scored the second most successes: 7 of 14.

**Table 4:** Amplification success for extractions, phase 1.

		Generic *rbcL* plant marker amplified for given method^1^				
Sample	Replicate^2^	Finnzymes	Epicentre	Gilbert	Japelaghi	MO BIO
ARE-A	A	−	−	−	−	−
	B	−	−	−	−	−
ARG	A	−	−	(+)	−	−
	B	−	−	+	−	−
CPR-A	A	+	(+)	+	−	+
	B	−	−	+	(+)	−
LUG	A	−	+	+	+	+
	B	−	+	+	+	+
PAR-A	A	−	−	+	+	−
	B	−	(+)	+	−	−
SAM	A	−	−	−	(+)	−
	B	−	−	−	−	−
VAD-A	A	(+)	+	(+)	+	(+)
	B	−	(+)	+	+	−
Amplification successes		2/14	6/14	10/14	7/14	4/14

1 + indicates a distinct band on 2% agarose gel, (+) indicates a faint band, and − indicates no band.

2 Amplifications for Finnzymes and Epicentre were conducted at (A) full strength and (B) 10% dilutions to test for enzymatic inhibition rather than two separate extractions of different seeds.

Amplification successes were compared using the generalized estimating equations function in PASW Statistics 18.0 [45]. This approach accommodates the presence of replicates for a given method and controls for success rates within each set of samples, even with limited numbers of samples. The Wald test found the best performing technique, the Gilbert method, to have significantly higher odds of amplifying the *rbcL* marker than the Finnzymes and MO-BIO techniques (p = 0.001 and 0.018, respectfully). The difference between the Gilbert method and the other two methods was not statistically significant (Epicentre, p = 0.061; Japelaghi, p = 0.273); however, qualitatively, it yielded stronger, more distinct PCR bands than the others.

**Figure 1:**
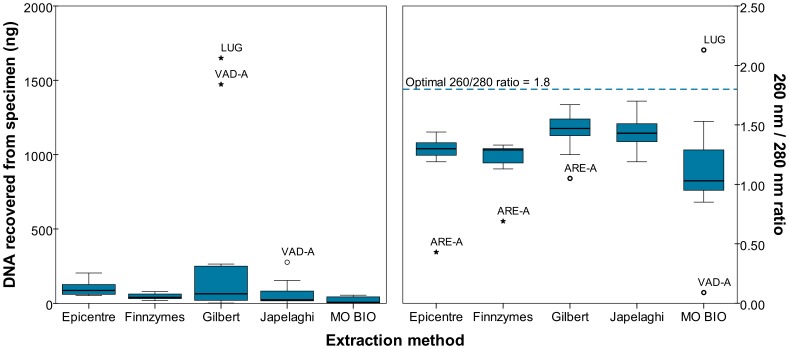
DNA yield and purity for extractions, phase 1.

None of the methods yielded amplifiable DNA from ARE-A, but this could be due to degradation of the sample (i.e. the endogenous DNA was shorter than the 138 bp *rbcL* marker). Therefore, DNA concentrations and purity readings for this sample are still considered germane. The mean amount of DNA for the Gilbert method was 304.5 ng, nearly triple the second highest value, 102.2 ng by Epicentre. After omitting the outliers shown in the left side of Figure 1, values were compared using a univariate generalized linear model (mixed model ANOVA) to control for differences between specimens. The model determined the method [F(4, 24) = 6.771, p = 0.001], specimen [F(6, 24) = 5.566, p = 0.001], and interaction between method and specimen [F(18, 24) = 9.607, p<0.001] to be statistically significant. Tukey’s HSD post-hoc test finds the Epicentre and Gilbert methods yield statistically significant greater amounts of DNA than other methods (p<0.001), but the difference between the two is not statistically significant (p = 0.885).

The right side of Figure 1 depicts the ratio of light absorbance at 260 and 280 nm, where a ratio of 1.8 is commonly considered to represent pure DNA [46]. None of the five methods consistently reached a ratio of 1.8, perhaps due to the low amount of aDNA in specimens, but the Gilbert method was the closest. After omitting the five outliers, an ANOVA test found statistical differences in the ratio of 260/280 between methods [F(4, 41) = 10.862, p<0.001], and Tukey’s HSD post-hoc test found the Gilbert method to have a statistically higher 260/280 ratio than the Finnzymes (p = 0.014) and MO BIO (p<0.001), but not the Epicentre (p = 0.116) or Japelaghi methods (p = 0.867).

#### Phase 2

As the Gilbert method performed the most consistently in phase 1, with the highest rate of successful amplification, the most DNA, and the purest eluates, it was promoted to more testing in phase 2. In terms of amplification success, all methods without C2/C3 solutions yielded PCR bands for SAF and VAD-B samples, and the Rohland method also produced a weak band for the CAS sample, as listed Table S2. Cloning and sequencing of the PCR bands showed that sequences for the SAF and VAD-B samples were identical to the expected sequence, or <2 bp different from the sequence, an error rate generally consistent with damaged DNA. None of the recovered sequences of CAS sample from the Rohland method were closer than 2 bp to the expected sequence and therefore likely represent contamination.

**Figure 2:**
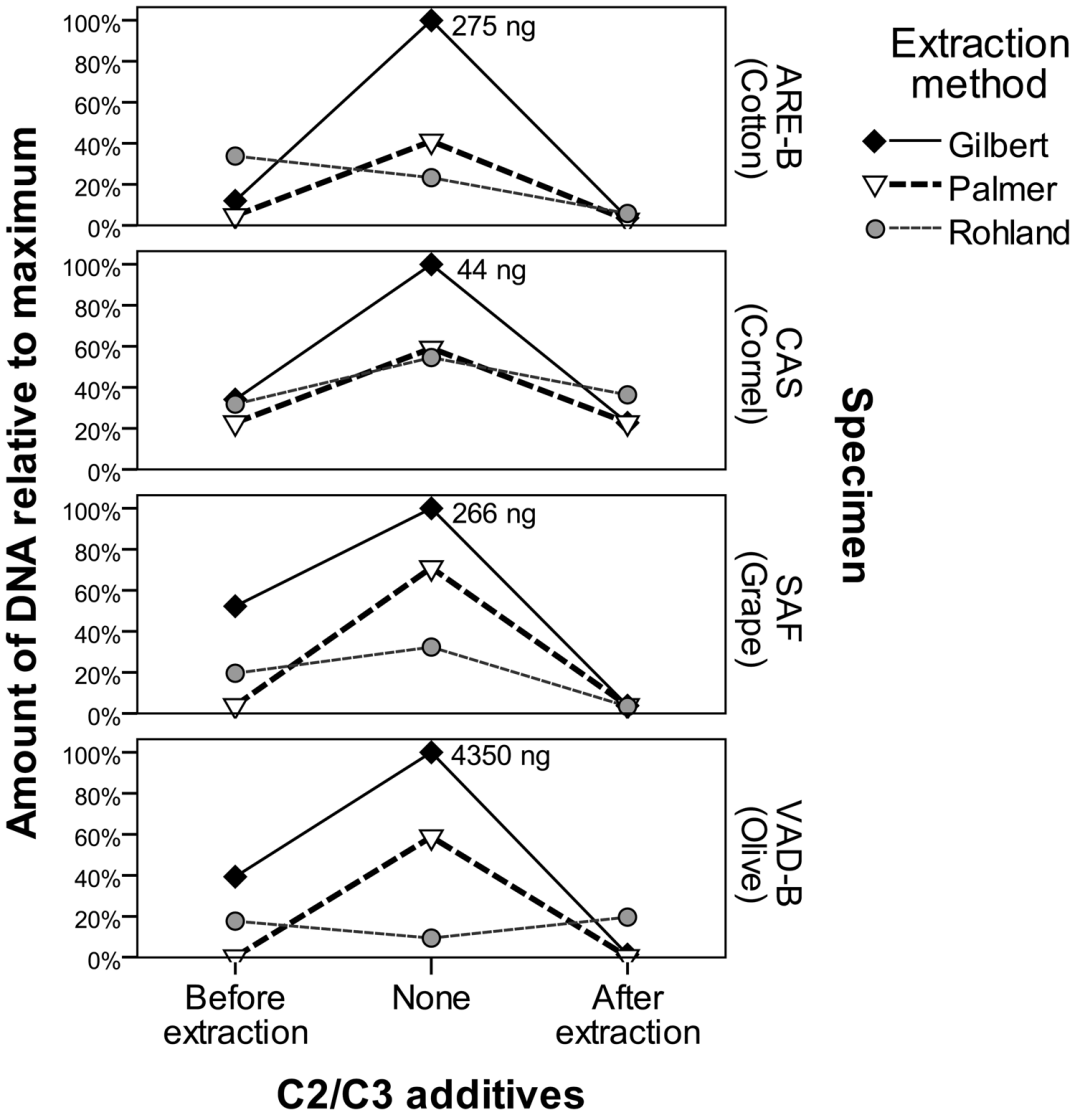
DNA yield from extractions, phase 2. Maximum amount of DNA recovered in each specimen listed by corresponding symbol.

The unmodified Gilbert method yielded more DNA than the other methods, and the addition of C2/C3 nearly always decreased DNA yield, as seen in Figure 2. To control for major differences in DNA recovery between specimens, DNA yield values were compared after logarithmic transformation. Log values were tested in a univariate generalized linear model controlling for differences in specimens, and found to have significant effects of extraction method [F(2, 28) = 3.563, p = 0.042], C2/C3 additives [F(2, 28) = 14.278, p<0.001], and specimen [F(3, 28) = 13.239, p<0.001]. Tukey’s HSD post-hoc identifies the addition of C2/C3 solutions before (p = 0.010) or after (p<0.001) an extraction to significantly reduce DNA recovery. An ANOVA test on the extractions not modified with C2/C3 solutions was significant for method [F(2, 6) = 10.109, p = 0.012] and specimen [F(3, 6) = 42.802, p<0.001]. The Gilbert method was found to recover significantly more DNA than the Rohland method (p = 0.010), but there was no significant difference between the Gilbert and the Palmer methods (p = 0.236).

The unmodified Gilbert and Palmer methods have statistically identical mean 260/280 ratios: 1.465 and 1.515, respectively. The Rohland method yielded ratios ranging from 1.10 to 4.87, likely due to low DNA content or residual particles from the silica extraction. When modified by C2/C3, the 260/280 ratios were not consistently brought closer to the ideal value of 1.8, as can be seen in Table S2. In all, there was no compelling evidence that the C2/C3 additions improved DNA purity, however, they certainly reduced DNA content.

#### Phase 3

The Gilbert and Palmer techniques were further tested in the final extraction phase, along with the Andersen sediment-style extraction. In terms of amplification success, the methods performed similarly: the Andersen and Palmer methods amplified six samples, while the Gilbert method amplified the same six as well as PAR-B. PCR was also tested without BSA, leading to the failure of nearly every reaction. The only samples amplifiable without BSA were THR (successful in all three methods) and VAD-B (a faint band in Palmer’s method). This finding may have important implications for the use of BSA in PCR on aDNA from non-charred archaeobotanical remains, as discussed below.

**Figure 3:**
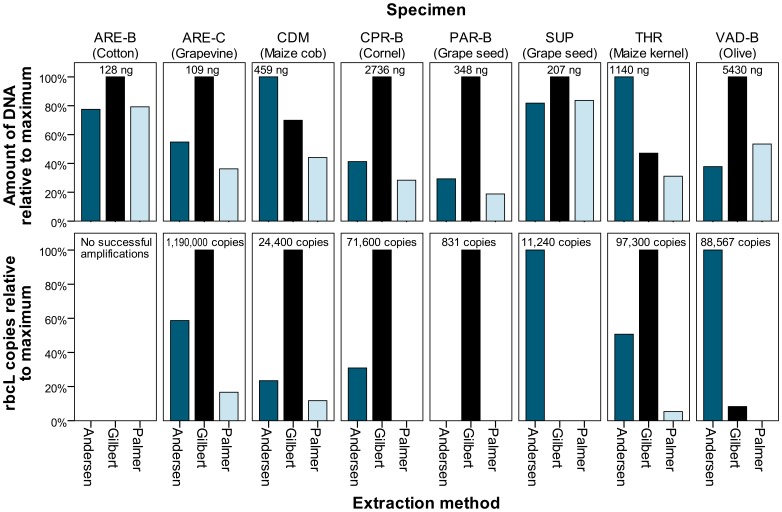
DNA yield and *rbcL* copies extracted during phase 3. DNA yield (top) calculated using a Qubit 1.0 Fluorometer and *rbcL* copies (bottom) determined by qPCR. Values are scaled to the maximum value of each sample, with the highest value listed above the corresponding bar. Missing bars in lower portion of figure indicates that a sample did not amplify in qPCR.

DNA purities were statistically identical, with mean 260/280 ratios of 1.527 (sd = 0.188), 1.558 (sd = 0.157), and 1.524 (sd = 0.245) for the Andersen, Gilbert, and Palmer methods, respectively. The amount of DNA recovered by the methods was more variable, as shown in top half of Figure 3. Mean DNA recovery was highest in the Gilbert method (1226.9 ng, sd = 1909.1), followed by the Andersen (651.1 ng, sd = 722.2) and Palmer (597.6 ng, sd = 968.6) methods. Log transformed DNA yields were tested in a univariate generalized linear model controlling for differences in specimens (mixed model ANOVA), and were found to have significant effects for extraction method [F(2, 14) = 6.539, p = 0.012] and specimen [F(3, 28) = 13.239, p<0.001]. Post-hoc testing with Tukey’s HSD test found the Gilbert method recovered a statistically significantly greater amount of DNA than the Palmer method (p = 0.007), but not the Andersen method (p = 0.184).

The number of copies of the *rbcL* gene recovered by each method varies dramatically between methods and samples. As seen in Figure 3, the number of *rbcL* copies as determined by qPCR does not perfectly reflect the amount of DNA measured on the Qubit fluorometer. This may indicate less pure eluates occasionally yield errant values. It could also be possible the methods differ in their ability to extract endogenous and exogenous DNA. To control for the wide large range of values, a logarithmic transformation was done, using log(x+1) to incorporate zero values. A mixed model ANOVA found the method [F(2, 14) = 4.707, p = 0.027] and specimen [F(7, 14) = 5.646, p = 0.003) to be significant factors in the number of recovered *rbcL* copies. Tukey’s HSD post-hoc test determined the Andersen method recovers significantly more *rbcL* copies than the Palmer method (p = 0.043), but there is not statistical difference between the Andersen and Gilbert methods (p = 0.995). Results provided by the Gilbert method are also found to differ from those provided by the Palmer method, but the differences are just beyond the threshold of statistical significance (p = 0.051).

### DNA Polymerases

#### Ability to overcome inhibition

The five polymerases demonstrated great variability in overcoming inhibition from substances found in ancient plant materials, as shown in Table 5. Without BSA additives, only Omni Klentaq and Phire Hot Start II were successful amplifying spiked tiger DNA in the presence of inhibitors, yielding PCR bands in reactions containing up to 1% of the ARE-B eluate. The addition of BSA enabled all polymerases to be functional in reactions containing at least 1% inhibiting substances. With BSA, AmpliTaq Gold, Omni Klentaq, and Phire overcame inhibition in at least one sample with 5% inhibitors. Omni Klentaq particularly exceled when BSA was added, successfully amplifying reactions containing 10% inhibiting solutions.

**Table 5:** Amplification of spiked DNA in the presence of inhibiting substances.

			Polymerase^1^				
BSA additive	Inhibiting solution	Amount of inhibitorin reaction	AmpliTaq Gold	Omni Klentaq	PfuTurbo C_x_	Phire	Phusion
No BSA	SPC	0%	+	+	+	+	+
		0.1%	−	+	−	+	−
		≥1%	−	−	−	−	−
	ARE-B	0%	+	+	+	+	+
		0.1%	−	+	−	+	−
		1%	−	(+)	−	(+)	−
		≥2.5%	−	−	−	−	−
BSA added	SPC	0%	+	+	+	+	+
		0.1%	+	+	+	+	+
		1%	+	+	+	+	+
		2.5%	+	+	−	+	−
		5%	(+)	+	−	(+)	−
		10%	−	+	−	−	−
		≥20%	−	−	−	−	−
	ARE-B	0%	+	+	+	+	+
		0.1%	+	+	+	+	+
		1%	+	+	+	+	+
		2.5%	+	+	(+)	+	(+)
		5%	−	+	−	+	−
		10%	−	+	−	−	−
		≥20%	−	−	−	−	−

1 + indicates a distinct band on 2% agarose gel, (+) indicates a faint band, and − indicates no band.

#### Compatibility with qPCR

**Figure 4:**
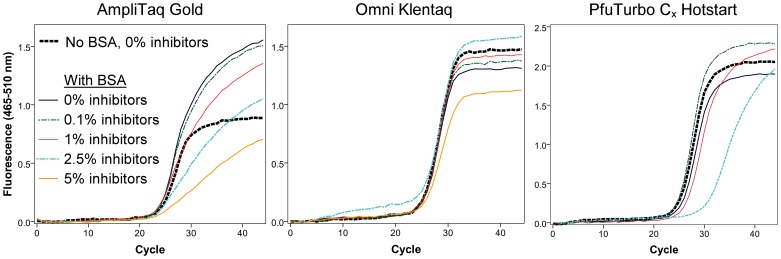
Compatibility of polymerases with qPCR. Inhibitory substances extracted from the SPC sample prevented amplification of spiked DNA in all reactions not including BSA, except for Omni Klentaq in 0.1% inhibitors (not shown). Unsuccessful amplifications, including PfuTurbo C_x_ Hotstart in 5% inhibitors, are not included in figure.

The three polymerases tested in qPCR behaved very differently when amplifying spiked DNA in the presence of BSA and inhibitors, as can be observed in Figure 4 and Table S3. The addition of BSA had a negative impact on the amplification curve in AmpliTaq Gold, but not the other polymerases. Increasing concentrations of inhibitors further reduced the slope of the amplification phase of AmpliTaq Gold reactions, and also affected PfuTurbo C_x_ Hotstart when inhibitors reached 2.5%. Conversely, Omni Klentaq was remarkably resilient to amplification inefficiencies due to inhibition.

#### Fidelity

The PfuTurbo C_x_ Hotstart polymerase was unable to amplify plant DNA in the LUG sample; therefore, 14 of the 15 possible combinations of specimens and polymerases were analyzed. Deep sequencing of the *rbcL* plant marker showed the vast majority of recovered sequences were consistent with the expected endogenous sequence, listed in Table S4. The entire dataset of sequencing reads is available online in Data S1–S14. All reads differing from the expected sequence by more than 3 bp were excluded from analyses, leaving 99.2%–99.9% of the original data for each case.

Three polymerases yielded a small number of sequences that could not be aligned to *rbcL* markers, shown in Table S4. Some of these were determined to be chimeras of amplicons. Notably, Omni Klentaq had a relatively high percentage of non-aligning reads. Additionally, Omni Klentaq was observed to occasionally yield DNA smears on agarose gels, a characteristic consistent with replication errors.

**Figure 5:**
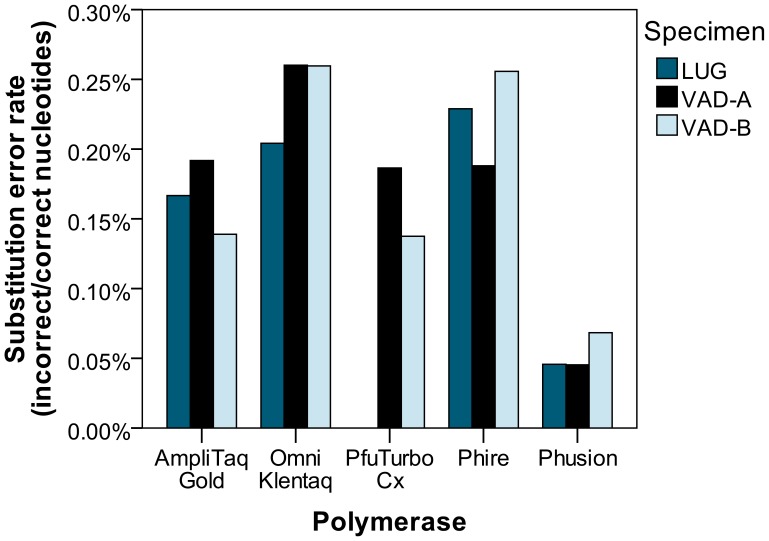
Overall substitution error rates on endogenous aDNA. Shorter bars represent fewer nucleotide misincorporations (higher polymerase fidelity). Sequencing reads that differed from the expected *rbcL* sequence by >3 nucleotide substitutions were omitted prior to tallying nucleotide calls and errors. As stated, the PfuTurbo C_x_ polymerase did not amplify the LUG sample.

Nucleotide substitution rates were calculated as the number of incorrect nucleotides divided by the number of correct nucleotides [47], listed in Table S5. Sequencing errors and DNA damage undoubtedly contribute to the overall error rate, but they are expected to be relatively constant across samples. As seen in Figure 5, Phusion polymerase had a consistently lower error rate than the other polymerases. A one-way ANOVA test found statistical differences in the error rates between polymerases [F(4, 9) = 20.022, p<0.001] and Tukey’s HSD post-hoc test found Phusion’s error rate to be significantly lower than the other polymerases (versus AmpliTaq Gold: p = 0.006, PfuTurbo C_x_: p = 0.015, and Omni Klentaq and Phire: p<0.001). Differences among the other polymerases were not statistically significant.

Phusion was also found to have the lowest error rates for nucleotide insertions and deletions, but several other polymerases had similar rates, as seen in Table S5. A one-way ANOVA test found statistically significant differences among the samples in nucleotide deletion rates [F(4, 9) = 3.976, p = 0.040], but not insertion rates [F(4, 9) = 2.031, p = 0.173]. Tukey’s HSD post-hoc test found the deletion rate in Phusion to be statistically different from that of AmpliTaq Gold (p = 0.025).

#### Compatibility with damaged DNA

**Figure 6:**
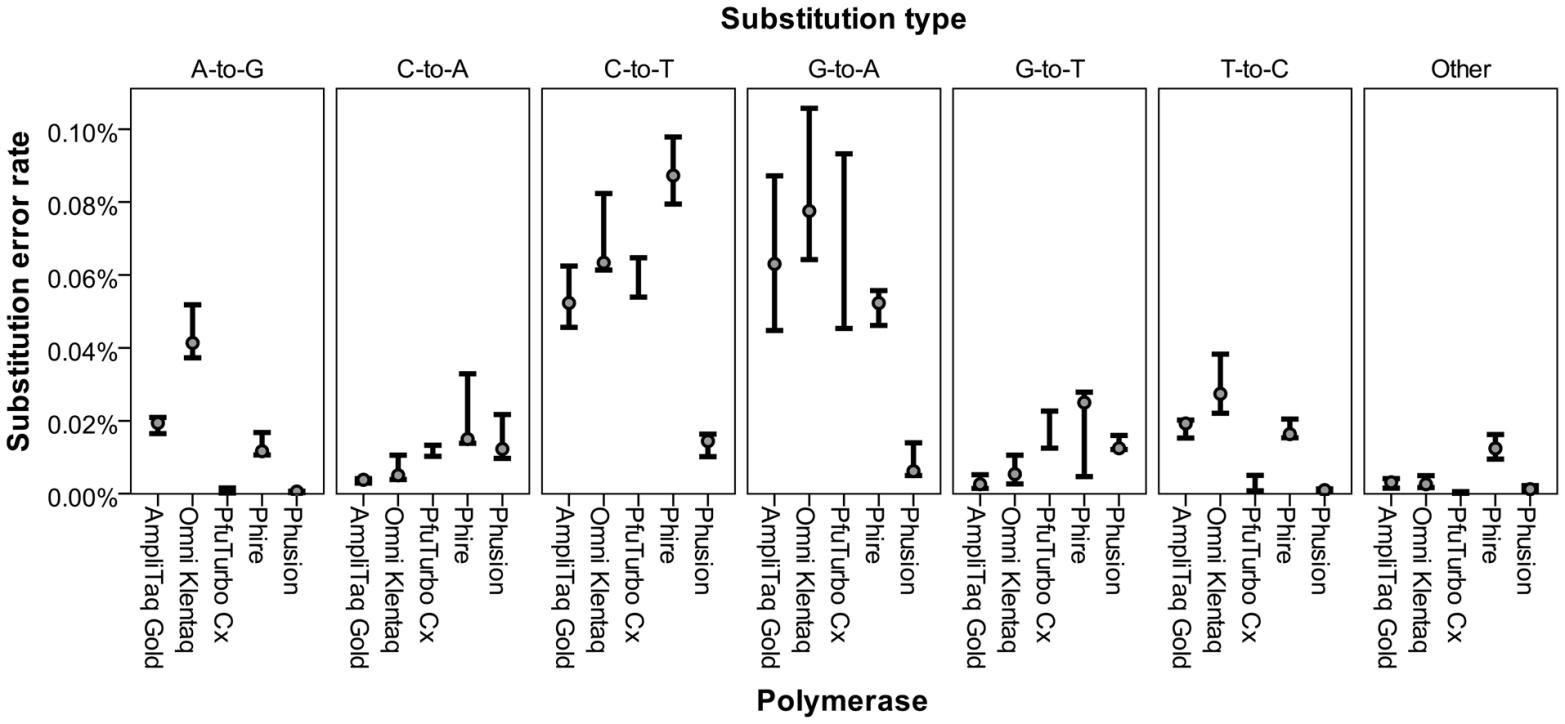
Error rates of most frequent substitution types. High-low chart depicts the maximum and minimum error rates within the three tested samples. Median values, represented by circles, are not included for PfuTurbo C_x_ because only two samples were amplified.

According to the manufacturer, Phusion polymerase is incompatible with uracil, causing DNA replication to stall. Conversely, PfuTurbo C_x_ is advertised as able to read uracil, resulting in an apparent C-to-T transition on the template strand and G-to-A transition on the complementary strand. Nucleotide substitutions rates in the other three polymerases were compared to those of Phusion and PfuTurbo C_x_ to determine if they follow similar patterns. As seen in Figure 6, Phusion has lower error rates in C-to-T and G-to-A transitions than the other polymerases. An ANOVA test on the error rates for individual samples found statistically significant differences in error rates for C-to-T [F(4, 9) = 30.846, p<0.001] and G-to-A [F(4, 9) = 7.045, p = 0.007] transitions. Tukey’s HSD post-hoc test on the C-to-T transitions found Phusion to have a statistically different error rate than the other polymerases (p≤0.002 for each pairwise comparison). Tukey’s HSD post-hoc test on the G-to-A transitions found Phusion to have a statistically different error rate than AmpliTaq Gold (p = 0.028), Omni Klentaq (p = 0.005), and PfuTurbo C_x_ (p = 0.034), but not Phire (p = 0.103). Overall, none of the polymerases tested have a pattern consistent with Phusion, indicating they pair uracil with adenine rather than stalling. The expanded dataset with error rates for all substitution types is available in Table S6.

## Discussion

These experiments provide a new perspective on how to extract and amplify endogenous DNA from non-charred archaeobotanical remains. Now that researchers are incorporating HTS technologies into the study of aDNA from ancient plant remains [19], [35], [48], these findings should prove especially useful, and may aid future research on critical issues surrounding plant evolution, domestication, and cultivation.

In order to fully profit from HTS of ancient remains, steps should be taken to optimize aDNA recovery. For archaeobotanical remains, these concerns are not trivial, because samples are often small and suboptimal approaches yield insufficient quantities of DNA, potentially leading to the destruction of samples for little or no gain. In the extraction experiments conducted here, the method that consistently performed the best is that described by Gilbert *et al.* [37]. While this method was developed by one of the authors, it was tested impartially, and found to recover more DNA with fewer co-extracted inhibiting substances than other techniques, even across a wide range of species and plant tissues. For previously untested archaeobotanical remains, it logically follows the Gilbert method provides the greatest chance for successful aDNA recovery. That being said, in the final round of testing, an extraction method developed for humic-rich sediments recovered more DNA from a few specimens, suggesting that it may be necessary to test a couple of methods for the most precious of samples. Of course, the insights garnered during this testing are limited to the set of extraction techniques used in the experiments. However, most methods commonly employed on ancient plant remains combine elements of the already tested approaches, so we do not anticipate such techniques to perform drastically differently.

It is interesting to consider how the best extraction methods compare to some others used in the field. For instance, the top two performing methods do not include cetyltrimethylammonium bromide (CTAB), a reagent used in many extraction methods on ancient plant remains, both charred [35], [49] and non-charred [48], [50]. CTAB is used to remove polysaccharides in modern plants [51], but contrary to conventional wisdom, it may not be necessary for non-charred archaeobotanical remains. Likewise, silica pellet extractions have been shown to excel at isolating aDNA from bones [15], but they did not perform as well on ancient plant samples in our testing. Unsurprisingly, commercial DNA extraction kits designed for use on freshly sampled modern plants were found to perform very poorly on ancient samples. Therefore, we would generally discourage aDNA researchers from using such kits on archaeobotanical remains, although similar kits have successfully yielded plant aDNA in some instances [52], [53].

Comparative testing of polymerases also yielded a number of important insights. One of the key findings is that no polymerase excels in all categories; rather, they have nuanced properties and should be selected with care, according to the goals and methods in a given research project, as outlined below. Some of the findings about particular polymerases have been reported [20], [54], but the results of these experiments can help select which polymerase to use in different circumstances.

One of the most commonly used polymerases in aDNA research, AmpliTaq Gold, was found to perform well in many categories, making it a good all-around polymerase. When used in conjunction with BSA, it can overcome moderate amounts of inhibition. Furthermore, it handles the most common form of nucleotide damage, cytosine deamination. Therefore, AmpliTaq Gold is well suited to amplify markers of interest in aDNA libraries, albeit with some reservation due to its replication error rate.

Phusion, a polymerase designed to have very high fidelity, was indeed found to have a much lower error rate than the other polymerases. However, Phusion is incompatible with uracil and stalls on damaged DNA templates. This is a critical concern for amplification of genetic markers or aDNA libraries, because Phusion will preferentially amplify non-damaged molecules, precisely those originating from modern contaminants. Therefore, some aDNA researchers, such as Green *et al.* [1], have devised a two-step amplification approach to retain damaged DNA but keep replication errors to minimum. First, a uracil-friendly polymerase, such as AmpliTaq Gold, is used to amplify over damaged nucleotides in a genetic marker or DNA library with a limited number of PCR cycles (10 cycles, for example). Then, in a second reaction, a high-fidelity polymerase, such as Phusion, is used to copy DNA with minimal errors, and reach the required number of DNA copies. Note that other strategies to deal with uracil in aDNA exist [55], but they are not based on polymerases and are therefore outside the realm of this article.

One of the most striking findings of the polymerase tests was the ability of Omni Klentaq to overcome inhibitory substances, consistent with findings on archaeological fish bone samples [20]. Even in high levels of inhibitory substances derived from non-charred ancient plant materials, like humic acids, Omni Klentaq successfully amplified spiked DNA when used with BSA. Without BSA, Omni Klentaq could still amplify DNA in the presence of low levels of inhibitors, a feat not matched by AmpliTaq Gold or Phusion. The significance of this property should not be overlooked, because enzymatic inhibition is not always recognized in the laboratory. For example, some DNA extracts in these studies contain inhibiting substances even though they lacked pigmentation. Omni Klentaq is also reliable in qPCR experiments where enzymatic inhibition may be encountered. Unlike AmpliTaq Gold, Omni Klentaq exhibits an exemplar qPCR amplification curve in the presence of BSA and inhibitors. Conversely, Omni Klentaq may have slightly lower fidelity than AmpliTaq Gold, and occasionally yields chimera amplicons, something not observed in other polymerases. Therefore, it is not an ideal polymerase to amplify libraries or other templates which will be sequenced. Nevertheless, it is an excellent choice for amplifying genetic markers in reticent samples and qPCR assays as it provides a safeguard against undetected enzymatic inhibition.

Another key discovery was that nearly all polymerases fail in the presence of inhibiting substances from non-charred archaeobotanical remains, unless BSA is added. In reactions without BSA, only Omni Klentaq and Phire could amplify spiked DNA, and even then, only the smallest concentrations of inhibitors could be overcome. When BSA was added to reactions containing small amounts of inhibiting substances, all polymerases were successful. This finding is even more important given the amplification tests from the third phase of extractions: irrespective of extraction method, AmpliTaq Gold nearly always failed to amplify endogenous plant markers unless BSA was added. While it might be assumed that plant-specific extraction protocols, such as those using CTAB, adequately purify DNA, they failed at virtually the same rate as other methods. Thus, we encourage adding BSA in PCR on non-charred archaeobotanical remains, contrary to the approach in most plant aDNA studies [39], [50], [56].

As we have not extracted charred archaeobotanical remains in these studies, we cannot directly test Giles and Brown’s [57] argument that BSA has no benefit for PCR on charred archaeobotanical remains and may reduce amplification success because DNA molecules become bound to BSA along with contaminants. However, it should be noted their study was based on artificially charred seeds and may not reflect the complexity of some archaeobotanical remains. For example, sediments adhering to charred cereals may contain humic acids that could inhibit PCR. Other things being equal, we suggest it is worth conducting PCR with BSA to ensure enzymatic inhibition does not lead to false negative results.

Some of the experimental methodology developed and refined over the course of this study could also provide guidance for future aDNA comparative experiments. For example, spectrophotometric detection of DNA in pigmented eluates was found to be occasionally misleading, so quantification of endogenous aDNA can be more reliably measured with qPCR and sequencing of PCR products. Testing of newly engineered polymerases will continue to be invaluable, and as demonstrated here, comparisons of fidelity and compatibility with damaged nucleotides can be successfully explored via HTS. Considering little is known about the inhibitory effects on polymerases and other enzymes used in the construction of DNA libraries, a similar set of experiments could be undertaken to optimize this fundamental step of HTS research.

## Conclusions

As foreseen by Palmer *et al.* [9], the future of plant aDNA research is very bright indeed. The introduction of high-throughput sequencing technologies allows geneticists to delve into ancient genomes in new and exciting ways. In fact, these technologies have already been tested on aDNA extracted from archaeobotanical remains [19], [35], [48]. However, in order for such studies to become more widespread and for the discipline to reach its full potential, it is critical the best available methods are used to extract, amplify, and analyze DNA from ancient specimens. For desiccated and waterlogged plant remains, this study is a step in that direction, and to that end, we strongly encourage fellow researchers to adopt the best performing extraction techniques, or at a minimum, conduct head-to-head comparisons with more familiar methods. Such experimentation will help advance plant archaeogenetics into a more fruitful discipline, yielding unprecedented understandings of plant evolution, domestication, and human-plant interactions.

## Supporting Information

Table S1 **PCR conditions for polymerases.**
(DOCX)

Table S2 **Extraction phase 2 data.**
(DOCX)

Table S3 **qPCR C_t_ values for polymerase inhibition testing.**
(DOCX)

Table S4 **Deep sequencing of** ***rbcL*** **markers to investigate polymerase fidelity.**
(DOCX)

Table S5 **Polymerase error rates.** Sequencing reads that differed from the expected *rbcL* sequence by >3 nucleotide substitutions were omitted prior to tallying nucleotide calls and errors.
(DOCX)

Table S6 **Specific substitution frequencies and corresponding error rate.** Sequencing reads that differed from the expected *rbcL* sequence by >3 nucleotide substitutions were omitted prior to tallying nucleotide calls and errors.
(DOCX)

Appendix S1 **Text with detailed extraction protocols, PCR information, DNA sequencing, and expanded results.**
(DOCX)

Data S1 **Deep-sequencing amplicon data of AmpliTaq Gold on LUG sample in FASTQ format.**
(FASTQ)

Data S2 **Deep-sequencing amplicon data of AmpliTaq Gold on VAD-A sample in FASTQ format.**
(FASTQ)

Data S3 **Deep-sequencing amplicon data of AmpliTaq Gold on VAD-B sample in FASTQ format.**
(FASTQ)

Data S4 **Deep-sequencing amplicon data of Omni Klentaq on LUG sample in FASTQ format.**
(FASTQ)

Data S5 **Deep-sequencing amplicon data of Omni Klentaq on VAD-A sample in FASTQ format.**
(FASTQ)

Data S6 **Deep-sequencing amplicon data of Omni Klentaq on VAD-B sample in FASTQ format.**
(FASTQ)

Data S7 **Deep-sequencing amplicon data of PfuTurbo C_x_ on VAD-A sample in FASTQ format.**
(FASTQ)

Data S8 **Deep-sequencing amplicon data of PfuTurbo C_x_ on VAD-B sample in FASTQ format.**
(FASTQ)

Data S9 **Deep-sequencing amplicon data of Phire on LUG sample in FASTQ format.**
(FASTQ)

Data S10 **Deep-sequencing amplicon data of Phire on VAD-A sample in FASTQ format.**
(FASTQ)

Data S11 **Deep-sequencing amplicon data of Phire on VAD-B sample in FASTQ format.**
(FASTQ)

Data S12 **Deep-sequencing amplicon data of Phusion on LUG sample in FASTQ format.**
(FASTQ)

Data S13 **Deep-sequencing amplicon data of Phusion on VAD-A sample in FASTQ format.**
(FASTQ)

Data S14 **Deep-sequencing amplicon data of Phusion on VAD-B sample in FASTQ format.**
(FASTQ)
