# Supplementary material for: Optimization of DNA Recovery and Amplification from Non-Carbonized Archaeobotanical Remains
Source: PLoS One. 2014 Jan 27;9(1):e86827. doi: 10.1371/journal.pone.0086827 (PMC3903575; doi:10.1371/journal.pone.0086827)
Supplement: Table S1 — PCR conditions for polymerases. (DOCX) [file pone.0086827.s001.docx]

Table S1. PCR conditions for polymerases.

| Polymerase | Vendor | PCR reagents | PCR conditions |
| --- | --- | --- | --- |
| AmpliTaq Gold | Applied Biosystems, Foster City, CA | 1 U polymerase, 0.4 µM primers, 0.2 mM dNTPs, 1X AmpliTaq Gold buffer, 2.5 mM MgCl_2_, 0-10 µL inhibiting solution | 4 min activation at 95˚C; 40 cycles of 20 s at 95˚C, 35 s at 58˚C, 60 s at 72˚C; and 7 min final extension at 72˚C |
| Omni Klentaq | DNA Polymerase Technology, St. Louis, MO | 0.25 µL polymerase, 0.2 µM primers, 0.2 mM dNTPs, 1X Omni Klentaq buffer, 0-10 µL inhibiting solution | 5 min activation at 94˚C, 40 cycles of 40 s at 94˚C, 60 s at 57˚C, 2 min at 68˚C |
| PfuTurbo C_x_ Hotstart | Agilent Technologies, La Jolla, CA | 0.5 µL polymerase, 0.2 µM primers, 0.2 mM dNTPs, 1X PfuTurbo Cx buffer, 1 µL DMSO, 0-10 µL inhibiting solution | 5 min activation at 95˚C; 40 cycles of 30 s at 95˚C, 30 s at 58˚C, 60 s at 72˚C; and 10 min final extension at 72˚C |
| Phire Hot Start II | Finnzymes (Thermo Fisher Scientific), Waltham, MA | 0.5 µL polymerase, 0.4 µM primers, 12.5 µL Plant Phire buffer, 0-10 µL inhibiting solution | 5 min activation at 98˚C; 40 cycles of 5 s at 98˚C, 5 s at 59˚C, 20 s at 72˚C; and 60 s final extension at 72˚C |
| Phusion Hot Start | Finnzymes (Thermo Fisher Scientific), Waltham, MA | 1X Phusion master mix, 0.4 µM primers, 0-10 µL inhibiting solution | 30 s activation at 98˚C; 40 cycles of 10 s at 98˚C, 30 s at 61˚C, 30 s at 72˚C; and 7 min final extension at 72˚C |
