# Supplementary material for: Optimization of DNA Recovery and Amplification from Non-Carbonized Archaeobotanical Remains
Source: PLoS One. 2014 Jan 27;9(1):e86827. doi: 10.1371/journal.pone.0086827 (PMC3903575; doi:10.1371/journal.pone.0086827)
Supplement: Table S2 — Extraction phase 2 data. (DOCX) [file pone.0086827.s002.docx]

Table S2. Extraction phase 2 data.

|  |  | Gilbert method | | Palmer method | | Rohland method | |
| --- | --- | --- | --- | --- | --- | --- | --- |
| Sample | C2/C3 addition | Amplification^[[1]](#footnote-1)^ | 260/280 ratio | Amplification | 260/280 ratio | Amplification | 260/280 ratio |
| ARE-B | Before | - | 1.40 | - | 1.25 | - | 2.05 |
|  | None | - | 1.37 | - | 1.33 | - | 1.99 |
|  | After | - | 1.35 | - | 1.22 | - | 2.07 |
| CAS | Before | - | 1.43 | - | 1.39 | (+) | 2.67 |
|  | None | - | 1.26 | - | 1.39 | (+) | 4.87 |
|  | After | - | 1.17 | - | 1.23 | (+) | 1.43 |
| SAF | Before | + | 1.44 | - | 1 | + | 2.55 |
|  | None | + | 1.50 | + | 1.57 | + | 2.74 |
|  | After | - | 1.25 | - | 1.06 | (+) | 3.22 |
| VAD-B | Before | + | 1.72 | - | 1.17 | + | 1.47 |
|  | None | + | 1.73 | + | 1.77 | + | 1.1 |
|  | After | + | 1.45 | - | 1.18 | - | 1.13 |

1. + indicates a distinct band for plant *rbcL* marker on 2% agarose gel, (+) indicates a faint band, and - indicates no band. [↑](#footnote-ref-1)
