# Supplementary material for: Optimization of DNA Recovery and Amplification from Non-Carbonized Archaeobotanical Remains
Source: PLoS One. 2014 Jan 27;9(1):e86827. doi: 10.1371/journal.pone.0086827 (PMC3903575; doi:10.1371/journal.pone.0086827)
Supplement: Table S3 — qPCR Ct values for polymerase inhibition testing. (DOCX) [file pone.0086827.s003.docx]

Table S3. qPCR C_t_ values for polymerase inhibition testing.

|  |  | C_t_ value ^[[1]](#footnote-1)^ | |
| --- | --- | --- | --- |
| Polymerase | Inhibitor level | No BSA | BSA added |
| AmpliTaq Gold | 0% | 24.78 | 24.58 |
|  | 0.1% | - | 24.58 |
|  | 1% | - | 24.22 |
|  | 2.5% | - | 24.20 |
|  | 5% | - | 24.17 |
| Omni Klentaq | 0% | 26.10 | 26.13 |
|  | 0.1% | 26.19 | 26.16 |
|  | 1% | - | 26.26 |
|  | 2.5% | - | 26.44 |
|  | 5% | - | 26.50 |
| PfuTurbo C_x_ Hotstart | 0% | 25.69 | 26.13 |
|  | 0.1% | - | 25.45 |
|  | 1% | - | 26.99 |
|  | 2.5% | - | 31.67 |
|  | 5% | - | - |

1. - indicates an unsuccessful amplification [↑](#footnote-ref-1)
