# Supplementary material for: Optimization of DNA Recovery and Amplification from Non-Carbonized Archaeobotanical Remains
Source: PLoS One. 2014 Jan 27;9(1):e86827. doi: 10.1371/journal.pone.0086827 (PMC3903575; doi:10.1371/journal.pone.0086827)
Supplement: Table S4 — Deep sequencing of rbcL markers to investigate polymerase fidelity. (DOCX) [file pone.0086827.s004.docx]

Table S4. Deep sequencing of *rbcL* markers to investigate polymerase fidelity.

|  |  | | Percent of reads mapped to expected *rbcL* sequence with *n* mismatches | | | | | | | |
| --- | --- | --- | --- | --- | --- | --- | --- | --- | --- | --- |
| Polymerase | Total number of reads | Non-*rbcL* sequences | 0 | 1 | 2 | 3 | 4 | 5 | 6–10 | 11–17 |
| AmpliTaq Gold | 17375 | 0 | 85.66 | 12.37 | 1.38 | 0.22 | 0.09 | 0.10 | 0.06 | 0.13 |
| Omni Klentaq | 15286 | 53 | 79.42 | 17.43 | 2.13 | 0.20 | 0.04 | 0.10 | 0.13 | 0.20 |
| PfuTurbo C_x_ Hotstart | 7007 | 0 | 84.40 | 14.27 | 1.17 | 0.06 | 0.03 | 0.03 | 0.03 | 0.01 |
| Phire Hot Start II | 23867 | 2 | 81.78 | 15.97 | 1.83 | 0.14 | 0.01 | 0.01 | 0.10 | 0.15 |
| Phusion Hot Start | 27173 | 8 | 95.05 | 4.42 | 0.20 | 0.01 | 0.11 | 0.07 | 0.01 | 0.10 |
