# Supplementary material for: Optimization of DNA Recovery and Amplification from Non-Carbonized Archaeobotanical Remains
Source: PLoS One. 2014 Jan 27;9(1):e86827. doi: 10.1371/journal.pone.0086827 (PMC3903575; doi:10.1371/journal.pone.0086827)
Supplement: Table S5 — Polymerase error rates. Sequencing reads that differed from the expected rbcL sequence by >3 nucleotide substitutions were omitted prior to tallying nucleotide calls and errors. (DOCX) [file pone.0086827.s005.docx]

Table S5. Polymerase error rates.

| Polymerase | Correct nucleotides | Substituted nucleotides (rate^[[1]](#footnote-1)^) | Inserted nucleotides (rate^1^) | Deleted nucleotides (rate^1^) |
| --- | --- | --- | --- | --- |
| AmpliTaq Gold | 1,642,165 | 2742 (0.1670%) | 532 (0.0324%) | 114 (0.0069%) |
| Omni Klentaq | 1,437,132 | 3405 (0.2369%) | 387 (0.0269%) | 51 (0.0035%) |
| PfuTurbo C_x_ Hotstart | 664,212 | 1176 (0.1771%) | 354 (0.0533%) | 22 (0.0033%) |
| Phire Hot Start II | 2,257,644 | 4788 (0.2121%) | 410 (0.0182%) | 89 (0.0039%) |
| Phusion Hot Start | 2,572,434 | 1319 (0.0513%) | 449 (0.0175%) | 36 (0.0014%) |

Sequencing reads that differed from the expected *rbcL* sequence by >3 nucleotide substitutions were omitted prior to tallying nucleotide calls and errors.

1. Error rates calculated as error count divided by number of correct nucleotides and converted to percentage [↑](#footnote-ref-1)
