# Supplementary material for: Optimization of DNA Recovery and Amplification from Non-Carbonized Archaeobotanical Remains
Source: PLoS One. 2014 Jan 27;9(1):e86827. doi: 10.1371/journal.pone.0086827 (PMC3903575; doi:10.1371/journal.pone.0086827)
Supplement: Table S6 — Specific substitution frequencies and corresponding error rate. Sequencing reads that differed from the expected rbcL sequence by >3 nucleotide substitutions were omitted prior to tallying nucleotide calls and errors. (DOCX) [file pone.0086827.s006.docx]

Table S6. Specific substitution frequencies and corresponding error rate.

|  | Number of observed substitutions for given polymerase (error rate^[[1]](#footnote-1)^) | | | | |
| --- | --- | --- | --- | --- | --- |
| Substitution | AmpliTaq Gold | Omni Klentaq | PfuTurbo C_x_ Hotstart | Phire Hot Start II | Phusion Hot Start |
| A-to-C | 8 (0.0005%) | 10 (0.0007%) | 0 (0.0000%) | 59 (0.0026%) | 2 (0.0001%) |
| A-to-G | 308 (0.0188%) | 604 (0.0420%) | 3 (0.0005%) | 267 (0.0118%) | 14 (0.0005%) |
| A-to-T | 3 (0.0002%) | 6 (0.0004%) | 0 (0.0000%) | 53 (0.0023%) | 0 (0.0000%) |
| C-to-A | 58 (0.0035%) | 83 (0.0058%) | 72 (0.0108%) | 382 (0.0169%) | 350 (0.0136%) |
| C-to-G | 13 (0.0008%) | 11 (0.0008%) | 1 (0.0002%) | 75 (0.0033%) | 14 (0.0005%) |
| C-to-T | 887 (0.0540%) | 1001 (0.0697%) | 416 (0.0626%) | 1970 (0.0873%) | 352 (0.0137%) |
| G-to-A | 1088 (0.0663%) | 1187 (0.0826%) | 558 (0.0840%) | 1140 (0.0505%) | 197 (0.0077%) |
| G-to-C | 14 (0.0009%) | 7 (0.0005%) | 1 (0.0002%) | 59 (0.0026%) | 6 (0.0002%) |
| G-to-T | 53 (0.0032%) | 77 (0.0054%) | 96 (0.0145%) | 344 (0.0152%) | 340 (0.0132%) |
| T-to-A | 7 (0.0004%) | 5 (0.0003%) | 1 (0.0002%) | 33 (0.0015%) | 14 (0.0005%) |
| T-to-C | 294 (0.0179%) | 398 (0.0277%) | 28 (0.0042%) | 370 (0.0164%) | 27 (0.0010%) |
| T-to-G | 9 (0.0005%) | 16 (0.0011%) | 0 (0.0000%) | 36 (0.0016%) | 3 (0.0001%) |

Sequencing reads that differed from the expected *rbcL* sequence by >3 nucleotide substitutions were omitted prior to tallying nucleotide calls and errors.

1. Error rates calculated as error count divided by number of correct nucleotides (found in Table S5) and converted to percentage [↑](#footnote-ref-1)
