# Supplementary material for: Optimization of DNA Recovery and Amplification from Non-Carbonized Archaeobotanical Remains
Source: PLoS One. 2014 Jan 27;9(1):e86827. doi: 10.1371/journal.pone.0086827 (PMC3903575; doi:10.1371/journal.pone.0086827)
Supplement: Appendix S1 — Text with detailed extraction protocols, PCR information, DNA sequencing, and expanded results. (DOCX) [file pone.0086827.s007.docx]

# Extraction method protocols with modifications

## Phase 1

**Epicentre extraction.** Plant samples were extracted using QuickExtract Plant DNA Extraction Solution (Epicentre, Madison, WI). Crushed seeds were placed in 300 µL of the extraction solution, incubated at 65°C for 6 minutes and 98°C for 2 minutes, and immediately cooled on ice. PCR was conducted using the solution in full concentration and 10% dilution.

**Finnzymes extraction.** Samples were extracted using the Finnzymes Phire Plant Direct PCR Kit (Thermo Fisher Scientific, Waltham, MA). Rather than following the “direct protocol,” DNA was extracted using the manufacturer’s optional “dilution protocol” for plant seeds. Cleaned and pulverized seeds were placed in 200 µL of dilution buffer (instead of the recommended 20 µL). Samples were briefly vortexed and incubated for 3 minutes at room temperature. The buffer was used as the template in PCR at full concentration and 10% dilution.

**Gilbert *et al.* extraction [1].** Cleaned and crushed samples were placed in a 1.5 mL microcentrifuge tube with 750 µL of digestion buffer consisting of: 10 mM tris(hydroxymethyl)aminomethane hydrochloride (Tris-HCl) (pH 8.0), 10 mM NaCl, 2% w/v sodium dodecyl sulfate (SDS), 5 mM CaCl_2_, 2.5 mM ethylenediaminetetraacetic acid (EDTA) (pH 8.0), 40 mM dithiothreitol (DTT), and 10% Proteinase K. All reagents were certified for molecular biology research. Samples were incubated overnight at 55°C. The following day, samples were equilibrated to room temperature and centrifuged at maximum speed for 5 minutes to pellet the plant tissue. The supernatant was placed in a new microcentrifuge tube and an equal volume of phenol was added. The samples were gently rotated for 5 minutes at room temperature and then centrifuged at maximum speed for 5 minutes. The aqueous layer was removed without disturbing protein-rich phases. An equal amount of phenol was added, and the rotation and centrifugation steps were repeated. The aqueous phase was collected and added to an equal volume of chloroform. The samples were shaken vigorously for 5 minutes and centrifuged for 5 minutes to separate the phases. The aqueous phase was collected and purified with a MinElute PCR Purification kit (Qiagen, Valencia, CA). Manufacturer directions were followed, except for the following modifications. The sample/PB binding buffer mixture was centrifuged at 6000 x *g*. It was necessary to conduct multiple rounds of centrifuging to pass the entire volume through a single spin column. The PE wash buffer was initially centrifuged for 1 minute at 10,000 x *g*. After disposing the flow-through, the spin column was centrifuged at maximum speed for 3 minutes to remove all traces of ethanol. 30 µL of EB buffer was added directly to the silica filter, and the spin column was incubated at 37°C for 10 minutes to increase yield. DNA was eluted into a new tube via centrifugation at maximum speed for 1 minute.

**Japelaghi *et al.* extraction [2].** DNA was extracted following Japelaghi *et al.*’s [2] instructions, with a few modifications to improve the recovery of short aDNA molecules. An extraction buffer consisting of 300 mM Tris-HCl (pH 8.0), 25 mM EDTA (pH 8.0), 2 M NaCl, 2% w/v polyvinylpyrrolidone (PVP), and 2% w/v CTAB was warmed to 65°C for 30 minutes. After warming the buffer, 2% v/v 2-mercaptoethanol was added. 900 µL of the prepared buffer was added to pulverized plant remains, and samples were incubated overnight at 55°C. The following day, samples were equilibrated to room temperature, and centrifuged at maximum speed for 5 minutes. The supernatant was collected and added to 750 µL chloroform-isoamyl alcohol. The solution was shaken for 5 minutes at room temperature and centrifuged for 5 minutes at maximum speed. The aqueous phase was collected and a second extraction with chloroform-isoamyl alcohol was conducted. DNA was purified using a MinElute PCR Purification kit (Qiagen, Valencia, CA) with the same modifications used in the Gilbert *et al.* (2004) extraction listed above.

**MO-BIO PowerLyzer PowerSoil DNA Isolation.** Cleaned and pulverized plant seeds were placed in a PowerLyzer glass bead tube with 750 µL of bead solution. After briefly vortexing, 60 µL of solution C1 was added and the sample was shaken in a FastPrep FP220A instrument (Qbiogene, Carlsbad, CA) for 45 seconds at 4 m/second. The samples were incubated at room temperature for 5 minutes and centrifuged at 10,000 x *g* for 30 seconds. The supernatant was transferred and the manufacturer’s protocols were followed for the addition and centrifugation of solutions C2, C3, C4, and C5. Elution was done with 30 µL of solution C6, and the spin column was incubated at 37°C for 10 minutes prior to collection via centrifugation.

## Phase 2

**MO BIO chemical additions.** The MO BIO C2 and C3 solutions (MO BIO Laboratories, Carlsbad, CA) are designed to precipitate humic acids and other inhibitors. The reagents were either added to digestion buffers after overnight incubations or to DNA eluates after the extraction. In either case, 250 µL of C2 was added to the DNA solution, incubated at 4° C for 5 minutes, and centrifuged at 10,000 x g for 1 minute; the process was then repeated using 200 µL of C3. Samples cleaned after overnight digestions proceeded directly to the extraction protocol. Samples cleaned with C2/C3 after extractions were concentrated to 30 µL using a MinElute spin column (Qiagen, Valencia, CA).

**Gilbert *et al.* extraction [1].** See phase 1.

**Palmer *et al.* extraction [3].** In the initial testing of Palmer *et al.*’s [3] method, a few modifications to the original protocol were included. Samples were place in 800 µL extraction buffer consisting of 0.1 M Tris-HCl (pH 8.0), 1.4 M NaCl, 20 mM EDTA (pH 8.0), and 2% w/v CTAB. Samples were shaken overnight at 37°C. The following day, samples were equilibrated to room temperature, and centrifuged at maximum speed for 5 minutes. The supernatant was collected and added to 750 µL chloroform-isoamyl alcohol. The solution was shaken for 5 minutes at room temperature and centrifuged for 5 minutes at maximum speed. The aqueous phase was collected and a second extraction with chloroform-isoamyl alcohol was conducted. Unlike the original protocol, DNA was purified using a MinElute PCR Purification kit (Qiagen, Valencia, CA) with the same modifications used in the Gilbert *et al.* (2004) extraction listed above.

**Rohland and Hofreiter extraction [4].** Archaeobotanical samples were processed using a silica extraction based on Rohland and Hofreiter’s [4] protocol. Silica pellets were prepared by mixing 6 g silicon dioxide in 50 mL molecular grade H_2_O. The tubes were vortexed and incubated for 1 hour. 48 mL of the supernatant was moved to a new tube and incubated for 5 hours. 43 mL of the supernatant was discarded and 60 µL of 37% HCl was added to the pellets. The pellets were resuspended and aliquoted into 100 µL volumes. Prepared pellets were used within a week.

Cleaned and pulverized samples were placed in 5 mL of digestion buffer consisting of 10 mM Tris-HCl (pH 8.0), 10 mM NaCl, 2% w/v SDS, 5 mM CaCl_2_, 2.5 mM EDTA (pH 8.0), 40 mM DTT, and 10% Proteinase K. Samples were incubated overnight at 55°C on a rotor. The next day, samples were centrifuged at 600 x *g* for 5 minutes and the supernatant was transferred to a new tube. The following were added to each sample: 50 µL silica pellet; 20 mL binding buffer consisting of 5 M Guanidium thiocyanate (GuSCN), 25 mM NaCl, 50 mM Tris (pH 8.0), 20 mM EDTA, and 20 mM N-lauryl Sarcosyl; and 100 µL 37% HCl. The solution was shaken at room temperature for 3 hours and centrifuged at 600 x *g* for 5 minutes. The supernatant was discarded and the pellet resuspended in 1 mL of buffer created by mixing 50 mL of the binding buffer from above with 250 µL 37% HCl. Samples were centrifuged at 4000 x *g* for 2 minutes and the supernatant discarded. The pellet was resuspended in 1 mL of 80% ethanol and centrifuged at 4,000 x *g* for 2 minutes. The supernatant was removed and the pellet was dried under a laminar flow hood. 60 µL of EB buffer (Qiagen, Valencia, CA) was added to the pellet, briefly vortexed, and incubated at 37°C for 15 minutes. The pellet was collected via centrifugation and the supernatant, with eluted DNA, was moved to a new tube.

## Phase 3

**Andersen extraction.** Kenneth Andersen, one of the authors of this article, developed this method to extract aDNA from sediments. Modifications to the method are still being tested and future revisions may lead to increased DNA yield or improved purity; thus, the method should be considered provisional.

Prior to extraction, a stock lysis buffer was made consisting of 68 mM N-Lauroylsarcosine sodium salt, 50 mM Tris-HCl (pH 8.0), 150 mM NaCl, and 20 mM EDTA (pH 8.0). Immediately before extraction, 0.43 mL 2-mercaptoethanol and 0.63 mL 1 M DTT were added to 11.44 mL of the above buffer.

Ancient plant remains were crushed and placed in a PowerLyzer glass bead tube (MO BIO Laboratories, Carlsbad, CA) with 800 µL of lysis buffer and 40 µL Proteinase K. The tube was vortexed vigorously for 1 minute to further pulverize the plant remains. Another 40 µL Proteinase K was added to the tube and then incubated overnight at 55°C while rotating. The following day, samples were equilibrated to room temperature, and centrifuged at 10,000 x *g* for 3 minutes to pellet the plant tissue. The supernatant was transferred to a clean 1.5 mL tube and 250 µL MOBIO solution C2 was added. Tubes were vortexed 5 seconds, incubated 5 minutes at 4°C, and centrifuged for 5 minutes at 10,000 x *g* to pellet inhibiting substances. The supernatant was transferred to a clean 1.5 mL tube and 200 µL MOBIO solution C3 was added. Tubes were vortexed 5 seconds, incubated 5 minutes at 4°C, and centrifuged for 5 minutes at 10,000 x *g* to pellet remaining inhibiting substances. The supernatant was placed in a new microcentrifuge tube and an equal volume of phenol was added. The samples were gently rotated for 5 minutes at room temperature and then centrifuged at maximum speed for 5 minutes. The aqueous layer was removed without disturbing protein-rich phases. An equal amount of phenol was added, and the rotation and centrifugation steps were repeated. The aqueous phase was collected and added to an equal volume of chloroform. The samples were shaken vigorously for 5 minutes and centrifuged for 5 minutes to separate the phases. The supernatant was transferred to a Millipore (Billerica, MA) 15 mL Amicon Ultra filter (30 kDa) for concentration and purification. The filter was centrifuged 10 minutes at 4,000 x *g*. The retained 50 µL of solution was washed with 500 µL EB buffer (Qiagen, Valencia, CA) and centrifuged 10 minutes at 4000 x *g*. The cleaning step was repeated for a total of two washes. 50 µL molecular grade H_2_O was added to the filter and incubated at room temperature for 10 minutes before collecting the purified DNA.

**Gilbert *et al.* extraction [1].** See phase 1.

**Palmer *et al.* extraction [3].** The published protocol was followed without modifications, insofar as the original protocol describes. Samples were place in 1000 µL extraction buffer consisting of 0.1 M Tris-HCl (pH 8.0), 1.4 M NaCl, 20 mM EDTA (pH 8.0), and 2% w/v CTAB. Samples were shaken for 24 hours at 37°C. The following day, samples were equilibrated to room temperature, and centrifuged at 13,000 x *g* for 5 minutes. The supernatant was collected and added to 950 µL chloroform-isoamyl alcohol. The solution was shaken for 5 minutes at room temperature and centrifuged for 5 minutes at 13,000 x *g*. The aqueous phase was collected and a second extraction with 850 µL chloroform-isoamyl alcohol was conducted. The supernatant was transferred to a Millipore (Billerica, MA) 15 mL Amicon Ultra filter (30 kDa) for concentration and purification. The filter was centrifuged 10 minutes at 4,000 x *g*. The retained 50 µL of solution was washed with 500 µL molecular grade H_2_O and centrifuged 10 minutes at 4,000 x *g*. The cleaning step was repeated for a total of two washes. 200 µL molecular grade H_2_O was added to the filter and incubated at room temperature for 10 minutes. The collected volume was further purified using a DNeasy silica spin column (Qiagen, Valencia, CA), following manufacturer’s directions. DNA was eluted with 100 µL of AE buffer.

# PCR information

## *rbcL* primers

The PCR primer set used to identify plants was designed by Poinar *et al.* [5]. The primers amplify a ~138 bp portion of the ribulose-bisphosphate carboxylase (*rbcL*) gene of the plastid genome, a fragment size which is short enough to be suitable for many ancient plant samples. The sequences of the forward and reverse primers are: 5'-GGCAGCATTCCGAGTAACTCCTC-3' and 5'-CGTCCTTTGTAACGATCAAG-3', respectively. The universal nature of the primers results in successful amplification in most angiosperms, and as this region of the *rbcL* gene is relatively diverse, phylogenetic affiliation of most plant families, as well as some genera, is possible.

## PCR for *rbcL* gene in all extraction phases

For all extractions except the Finnzymes method, 25 µL PCR reactions were conducted using a standardized amount of template DNA (usually 1 μL), 1 U AmpliTaq Gold (Applied Biosystems, Foster City, CA), 1X AmpliTaq Gold buffer, 2.5 mM MgCl_2_, 200 µM dNTPs, 400 nM primers, and 0.8 mg/mL bovine serum albumin (BSA). To compensate for extractions with different elution volumes, template volume in PCR was varied accordingly. For example, in extraction phase 3, the Gilbert extractions resulted in 30 μL of template DNA and the Andersen and Palmer extractions produced 100 μL of template; for PCR of the Gilbert samples, 1 μL was used, while 3.33 μL were used for the other two. PCR cycling conditions were as follows: 95.0°C for 4 minute enzyme activation, 40 cycles of 95.0°C for 20 seconds, 56.0°C for 35 seconds, 72.0°C for 45 seconds, and a final 7 minutes at 72.0°C. PCR for the Finnzymes extraction was conducted in 50 µL reactions using 1.25 µL of template DNA, 1 µL of the supplied Phire polymerase, 1X Phire PCR buffer, and 500 nM primers. PCR cycling conditions were as follows: 98.0°C for 5 minutes, 40 cycles of 98.0°C for 5 seconds, 60.0°C for 60 seconds, 72.0°C for 20 seconds, and a final 1 minute at 72.0°C. PCR for the Epicentre and Finnzymes methods was done using 100% and 10% dilution of the template DNA.

## qPCR assay for quantifying DNA in extraction phase 3

A real-time quantitative PCR (qPCR) assay was used to determine the amount of plant DNA in samples. The assay amplified the universal *rbcL* marker, as described above, and included negative controls as well as a series of DNA standards ranging from 10^8^ to 10 *rbcL* template molecules per reaction. By testing known concentrations of *rbcL* amplicons, it is possible to calculate the number of template *rbcL* molecules in each extract. qPCR was performed on a Roche LightCycler 480 Real-time PCR System using dye that fluoresces in the presence of double-stranded DNA. The assay was conducted in 25 µL reactions with 1 µL of template DNA, 1 U AmpliTaq Gold polymerase, 1X AmpliTaq Gold buffer, 2.5 mM MgCl_2_, 0.2 mM dNTPs, 0.4 µM primers, 0.8 mg/mL BSA, and 1 µL 1X SYBR Green/ROX mix (Invitrogen, Carlsbad, CA). Cycling conditions for qPCR were: 95.0°C for 10 minutes, 50 cycles of 95.0°C for 30 seconds and 54.0°C for 1 minute, followed by a melting curve. The cycle threshold (C_t_) values and amplicon copy numbers were determined by the LightCycler software

## PCR inhibition testing of polymerases

DNA extracts from two ancient plant samples were used as inhibiting substances in which to test five polymerases, listed in Table 2. Both samples were extracted using a Qiagen DNeasy Plant Mini kit (Valencia, CA), and experimentation demonstrated the darkly pigmented eluates readily inhibited amplification. PCR was conducted in 25 µL reactions containing 0, 0.025, 0.25, 0.625, 1.25, 2.5, 5, or 10 μL of one of inhibiting solutions, respectively 0, 0.1, 1, 2.5, 5, 10, 20, and 40% of the total volume.

An exogenous DNA marker was spiked into samples to ensure all reactions had the same template concentration. The 72 bp endogenous marker is a portion of the tiger (*Panthera tigris*) mtDNA D-loop, and has been used in other aDNA experiments because it is similar in length to what is commonly found in ancient samples [6]. Approximately 1000 copies of the marker were added to each reaction, along with forward and reverse primers. The primer sequences which amplify the marker are F: 5'-TCCAATCCTTCAACTTTCTCA-3' and R: 5'- ACAGTTATGTGTGATCATGGGC-3'. Primer concentrations and PCR conditions are described in Table S1.

Amplification success was determined by visualizing PCR amplicons on a 2% agarose gel stained with GelRed dye (Biotium, Hayward, CA).

## qPCR inhibition testing

Following the conventional PCR amplification of spiked DNA with varying levels of inhibitors, three polymerases were also tested in qPCR. Each 25 µL reaction contained 1000 copies of spiked tiger DNA, the reagents listed in Table S1, 1 µL of SYBR Green/ROX mix (Invitrogen, Carlsbad, CA), and a volume of pigmented eluates of the SPC grape seed sample representing one of the following concentrations in the reaction: 0%, 0.1%, 1%, 2.5%, and 5%. qPCR was performed on a Roche LightCycler 480 using thermal conditions suitable for the three polymerases: enzyme activation 10 minutes at 95˚C, 45 cycles of 30 seconds at 95˚C, 1 minute at 56˚C, and 1 minute at 72˚C, followed by a melt curve. C_t_ values were determined by the LightCycler software using the second derivative maximum method and high sensitivity algorithm.

# DNA sequencing

## Sanger Sequencing of *rbcL* products

For extraction phases 2 and 3, successful amplifications of the *rbcL* marker were sequenced to determine if endogenous DNA was recovered. PCR amplicons were cloned using a TOPO TA cloning kit (Invitrogen, Carlsbad, CA) and commercially sequenced by Macrogen Europe (Amsterdam).

## Deep sequencing of *rbcL* products

To study polymerase fidelity and compatibility with aDNA damage, *rbcL* markers were amplified using different polymerases and subsequently sequenced on a high-throughput sequencing platform. By using HTS on PCR amplicons instead of traditional bacterial cloning, deep sequencing yields thousands of copies of a locus, greatly increasing sample sizes, and permitting the discovery of rare variants, or in this case, observing the frequency of polymerase errors.

Three ancient plant samples were tested, as indicated in Table 2; all samples were extracted using the Gilbert method. PCR was conducted in 25 µL reactions containing 1 µL of template aDNA, primers targeting the *rbcL* plant marker, and the reagents listed in Table S1. PCR was conducted following the reagents and temperatures listed in Table S1.

Primers were modified to include a sample-specific 5’ index sequence [7] and to be compatible with the Roche/454 Genome Sequencer FLX platform (so-called Fusion primers). In this way, amplicons are immediately ready for sequencing without normal DNA library building steps. The set of indexed amplicons were pooled and size selected on a 2% agarose gel to remove residual primers and minor DNA smears. The processed pool was sequenced on a single lane of the GS FLX platform.

# Expanded Results

## Extraction comparisons

**Phase 1.** A separate set of extractions were tested which combined elements of the Japelaghi and Gilbert methods, by adding PVP and increasing salt concentrations in the Gilbert digestion buffer and adding Proteinase K to the Japelaghi method. None of the permutations demonstrated improved amplification efficiency or DNA yields (data not shown).

**Phase 2.** None of the sequences yielded by the Rohland method on the CAS sample perfectly matched the expected sequence. The sequences most similar to the expected *Cornus* sequence were 2–3 bp different, represented by 10 of the 23 recovered sequences. However, these sequences are identical to *Platanus orientalis* (Oriental plane). The remaining 13 *rbcL* sequences from the *Cornus* sample are most similar to the Anacardiaceae (Sumac) family, with 6 sharing 100% identity, and all different from *Cornus* by 6–9 bp. Therefore, even if the Rohland method would be able to extract endogenous DNA not recovered by the other two methods, it may do so at the cost of introducing contamination, likely during the extensive steps required for silica pellet preparation.

**Phase 3.** Cloning and Sanger sequencing of successful amplicons revealed that most PCR products matched the expected sequences. Only the CDM maize cob and SUP waterlogged grape seed had exogenous sequences. Of the SUP sequences, 86% were endogenous, the remainder consistent with the Oleaceae family, perhaps cross-contamination. For CDM, all sequences were exogenous, with matches to rosids and asterids. No trends were observed where one method consistently yielded more endogenous DNA than the other methods.

## DNA polymerases

**Compatibility with qPCR.** For each polymerase, the reaction conducted with neither BSA nor inhibitors serves as the control and exhibits the expected curve: a brief exponential amplification phase followed by a plateau. While there are slight differences in C_t_ values between polymerases for the control condition, shown in Table S3, the consistency within a given polymerase is more important because qPCR assays are normally conducted on a set of samples and standards using a single polymerase, so all reactions would be subjected to the same conditions.

The way in which BSA leads to changes in the amplification curve in AmpliTaq Gold is not totally understood; however, in normal PCR amplifications with AmpliTaq Gold and BSA, we frequently observe an opaque residue created during the heating and cooling cycles, perhaps the result of an interaction between BSA and components of the AmpliTaq Gold buffer. This white liquid may interfere with the qPCR light detection system, apparently intensifying the fluorescence. Regardless, this sort of curve is undesirable because measurements of the C_t_ values are less accurate.

While changes in plateau levels are not anticipated to affect quantification of DNA, the consistency of C_­t_ values in the presence of different levels of inhibitors is paramount. For each polymerase, there was no drastic change in C_­t_ values when BSA was added, as can be seen in Table S3; PfuTurbo C_x_ Hotstart had the greatest change, an increase of 0.44 cycles, thus less spiked tiger DNA being sensed. With higher levels of inhibitors, PfuTurbo C_x_ Hotstart had increasingly larger C_t_ values when inhibitors were 1% and 2.5%, and ultimately failed to amplify with 5% inhibitors. The other polymerases only slightly deviated from the control values when inhibitors were added: C_­t_ values increased up to 0.40 cycles in Omni Klentaq and decreased as much as 0.61 cycles in AmpliTaq Gold. Even though the data are limited, Omni Klentaq appears to have a slight advantage over AmpliTaq Gold, with a smaller standard deviation in C_­t_ values for reactions with BSA (0.166 vs. 0.211) and BSA values closer to the control (mean difference: 0.198 vs. -0.430 cycles). A one-sample T-test found the C_­t_ values for inhibited AmpliTaq Gold reactions to be statistically different from the control [*t*(4)=-4.563, p = 0.010], but the test does not quite support the conclusion for differences in the Omni Klentaq reactions [*t*(4)=2.674, p = 0.056].

**Fidelity.** While some endogenous reads should slightly differ from the expected sequence due to polymerase errors and aDNA damage, a small number of deep sequencing reads differed from the expected motif by 9–17 bp, as can be observed in Table S4. These reads match sequences of *Quercus* spp., *Magnolia* spp., and members of the Poaceae family, among others, indicating a minor degree of contamination from distantly related species. Other reads differed from the expected sequence by 4–5 bp, matching known sequences for *Cryptocarya* spp., *Crepis* spp., and members of the Apiaceae family. These most likely represent contamination by closely related species, a hypothesis supported by the declining frequencies of 1–3 bp mismatches seen in Table S4. Therefore all reads differing from the expected sequence by more than 3 bp were excluded from fidelity analyses, still retaining 99.2%–99.9% of the original data for each case. It should be noted that some other divergent sequences may be actually be endogenous, but originating in another portion of the genome. For example, a sequence recovered from the LUG sample was 15 bp different from the expected grape *rbcL* marker, but its top match in the NCBI-nt database is a *Vitis* shotgun sequencing read. It is plausible this represents a poorly characterized insertion of the *rbcL* marker into the nuclear or mitochondrial genome.

Identifying the number of nucleotide substitutions caused by polymerase errors is a complicated endeavor in this study because “mistakes” may also originate from damaged aDNA molecules or inherent errors of the HTS platform. A wide range of experimentally determined error rates have been reported for the GS FLX: meta-analysis of different studies by Niu *et al.* [8] found an average substitution error rate of 0.130%, with values ranging 0.026%–0.388%. The difference in rates is contingent upon the nature of the DNA molecules, as the platform makes increasingly frequent errors with long homopolymers [9] and read lengths >300 bp [10]. Given that the *rbcL* amplicon is only 138 bp and has one or two small homopolymer stretches (4 consecutive guanines in both grapes and olive plus 3 consecutive cytosines in grapes), it would not be surprising if the sequencing error rate was less in this study than the mean overall rate reported in other publications—in fact, Phusion was found to have an average error rate of 0.0513%. Forms of aDNA damage, including cytosine deamination, undoubtedly contribute to the substitution errors, but it is again difficult to reach a precise number. While it is possible to use shotgun sequencing data from aDNA to find characteristic damage at the 5' and 3' ends of aDNA molecules [11,12], quantifying the total number of errors in PCR amplicons due to aDNA damage is not well established and would take more experimentation before it could be confidently employed. Instead of estimating errors originating from the sequencing platform and damaged aDNA molecules, they were instead assumed to be relatively constant because the same samples were tested on a single sequencing run. Thus, samples should have a relatively constant underlying error rate, but differences beyond this baseline should be due to differences in polymerase fidelity. The substation, insertion, and deletion error rates for each polymerase are listed in Table S5.

**Compatibility with damaged DNA.** To accurately identify C/G-to-T/A transitions caused by polymerases in the sequencing data, it would be ideal to know the baseline error rates of the Roche/454 GS FLX for each possible substitution. This concern is irrelevant to most sequencing projects because the exact type of substitution errors is generally inconsequential and errors are ignored as a whole. Such error biases have been characterized on the Illumina HTS platforms [13], but these data are not available for the GS FLX. So, instead, the rates of different nucleotide substitutions were compared to see if they were more consistent with patterns seen in Phusion or PfuTurbo C_x_, advertised as stalling on and reading through uracil, respectively.

As discussed in the manuscript and observable in Figure 6 and Table S6, the C/G-to-T/A error rates of AmpliTaq Gold and Omni Klentaq are indistinguishable from those of PfuTurbo C_x_, and it is therefore reasonable to assume they handle uracil in a similar fashion. This is also consistent with findings that polymerases in the *taq* family can be used to read through aDNA damage [14]. The patterns seen in Phire are more challenging to interpret, as it has the highest rate of C-to-T transitions and the second lowest rate of G-to-A transitions; however, if the misincorporations were damage-related, the rates would be expected to be more similar, given the complementary nature of the errors [15]. It is probable that Phire can read over uracil, but it may also have a highly biased error rate for C-to-T transitions.

It should be noted that each individual polymerase may have proclivities to specific types of substitution fidelity errors, potentially comprising some of the substitutions assumed to be due to damage. For example, Omni Klentaq makes A-to-G and T-to-C misincorporations more often than the other polymerases, as shown in Figure 6. Theoretically, error biases could mimic DNA damage patterns, although the consistency in C-to-T and G-to-A error rates suggests the above interpretations are accurate. Nevertheless, knowledge of biases toward or against specific substitution errors may be helpful to identify common polymerase errors or when planning how to sequence certain loci, such as GC-rich regions.

# References

1. Gilbert MTP, Wilson AS, Bunce M, Hansen AJ, Willerslev E, et al. (2004) Ancient mitochondrial DNA from hair. Curr Biol 14(12): R463–R464.

2. Japelaghi R, Haddad R, Garoosi G (2011) Rapid and Efficient Isolation of High Quality Nucleic Acids from Plant Tissues Rich in Polyphenols and Polysaccharides. Mol Biotechnol 49: 1–9.

3. Palmer SA, Moore JD, Clapham AJ, Rose P, Allaby RG (2009) Archaeogenetic Evidence of Ancient Nubian Barley Evolution from Six to Two-Row Indicates Local Adaptation. PLoS ONE 4(7): e6301.

4. Rohland N, Hofreiter M (2007) Comparison and optimization of ancient DNA extraction. BioTechniques 42(3): 343–352.

5. Poinar HN, Hofreiter M, Spaulding WG, Martin PS, Stankiewicz BA, et al. (1998) Molecular Coproscopy: Dung and Diet of the Extinct Ground Sloth *Nothrotheriops shastensis*. Science 281(5375): 402–406.

6. Andersen K, Bird KL, Rasmussen M, Haile J, Breuning-Madsen H, et al. (2012) Meta-barcoding of 'dirt' DNA from soil reflects vertebrate biodiversity. Mol Ecol 21(8): 1966–1979.

7. Binladen J, Gilbert MTP, Bollback JP, Panitz F, Bendixen C, et al. (2007) The Use of Coded PCR Primers Enables High-Throughput Sequencing of Multiple Homolog Amplification Products by 454 Parallel Sequencing. PLoS ONE 2(2): 197.

8. Niu B, Fu L, Sun S, Li W (2010) Artificial and natural duplicates in pyrosequencing reads of metagenomic data. BMC Bioinformatics 11(1): 187.

9. Luo C, Tsementzi D, Kyrpides N, Read T, Konstantinidis KT (2012) Direct Comparisons of Illumina vs. Roche 454 Sequencing Technologies on the Same Microbial Community DNA Sample. PLoS ONE 7(2): e30087.

10. Gilles A, Meglécz E, Pech N, Ferreira S, Malausa T, et al. (2011) Accuracy and quality assessment of 454 GS-FLX Titanium pyrosequencing. BMC Genomics 12(1): 245.

11. Ginolhac A, Rasmussen M, Gilbert MTP, Willerslev E, Orlando L (2011) mapDamage: testing for damage patterns in ancient DNA sequences. Bioinformatics 27(15): 2153–2155.

12. Jónsson H, Ginolhac A, Schubert M, Johnson PLF, Orlando L (2013) mapDamage2.0: fast approximate Bayesian estimates of ancient DNA damage parameters. Bioinformatics 29(13): 1682–1684.

13. Dohm JC, Lottaz C, Borodina T, Himmelbauer H (2008) Substantial biases in ultra-short read data sets from high-throughput DNA sequencing. Nucleic Acids Res 36(16): e105.

14. Hofreiter M, Jaenicke V, Serre D, von Haeseler A, Pääbo S (2001) DNA sequences from multiple amplifications reveal artifacts induced by cytosine deamination in ancient DNA. Nucleic Acids Res 29(23): 4793–4799.

15. Gilbert MTP, Binladen J, Miller W, Wiuf C, Willerslev E, et al. (2007) Recharacterization of ancient DNA miscoding lesions: insights in the era of sequencing-by-synthesis. Nucleic Acids Res 35(1): 1–10.
